# Developing the novel bioinformatics algorithms to systematically investigate the connections among survival time, key genes and proteins for Glioblastoma multiforme

**DOI:** 10.1186/s12859-020-03674-4

**Published:** 2020-09-17

**Authors:** Yujie You, Xufang Ru, Wanjing Lei, Tingting Li, Ming Xiao, Huiru Zheng, Yujie Chen, Le Zhang

**Affiliations:** 1 College of Computer Science, Sichuan University, Chengdu, 610065 China; 2 Department of Neurosurgery, Southwest Hospital, Third Military Medical University, Chongqing, P.R. China; 3 College of Mathematics and Statistics, Southwest University, Chongqing, 400715 P.R. China; 4 School of Computing, Ulster University, Coleraine, Londonderry, Northern Ireland, UK

**Keywords:** Glioblastoma multiforme (GBM), Survival analysis, Adipocyte enhancer-binding protein 1 (AEBP1), Data mining, mTOR pathway

## Abstract

**Background:**

Glioblastoma multiforme (GBM) is one of the most common malignant brain tumors and its average survival time is less than 1 year after diagnosis.

**Results:**

Firstly, this study aims to develop the novel survival analysis algorithms to explore the key genes and proteins related to GBM. Then, we explore the significant correlation between *AEBP1* upregulation and increased *EGFR* expression in primary glioma, and employ a glioma cell line LN229 to identify relevant proteins and molecular pathways through protein network analysis. Finally, we identify that *AEBP1* exerts its tumor-promoting effects by mainly activating mTOR pathway in Glioma.

**Conclusions:**

We summarize the whole process of the experiment and discuss how to expand our experiment in the future.

## Background

Glioblastoma multiforme (GBM) is one of the most common malignant brain tumors and its average survival time is less than 1 year after diagnosis [1]. The occurrence of GBM is considered as a complicated biological phenomenon with multiple external simulating factors, genes and stages. The major challenge in the field is to translate the almost unique progress in deciphering the highly complex molecular genetic nature of GBM into advances that allow for better prognosis rate and survival of affected patients [2].

Currently, cancer researchers usually employ the survival analysis to explore genes that are closely related to GBM [2–5]. For example, Xia et al. [2] developed a CoxSisLasso survival analysis algorithm to identify gene signature for GBM by processing P> > N (the dimension of the factors P are less than the number of samples N) type of data [2]. However, since CoxSisLasso survival analysis algorithm does not consider the interactions among genes, its predictive accuracy is very limited. Therefore, we propose our first research question: Can we develop a more efficient survival analysis algorithm to explore the gene signature of GBM for P> > N type of data?

Secondly, current commonly used genomics [6] or proteomics [7] biotechnologies have limitations in quantitatively measuring gene expression and protein contents, which prevent us from developing the precise mathematical model. Thus, we propose our second research question: In order to explore the key proteins by using quantitative gene/protein data, can we employ cutting-edge biotechnologies, such as the CRISPR(clustered regularly interspaced short palindromic repeat sequences) [7–9] to knock out the key gene and then use RPPA(reverse phase protein arrays) [10–15] to high-throughput screen the corresponding protein data?

Thirdly, although a few GBM studies ([2, 3, 16–22]) considered the multi-scale data from intracellular, cellular and tissue scales, they neither develop the high efficient computational biology algorithm nor use experimental data to validate their findings in genomic and proteomic level. Thus, we propose our third research question: can we build a precise multi-scale mathematical model that can be used to understand the origin of the GBM from systematic view?

This study aimed to address the above research questions and the following novel approaches have been taken: (1) Firstly, we improved previous additive survival analysis algorithm(CoxSisLasso) [2, 9, 23] to investigate the key genes for GBM survival by considering genes’ interaction as well as used immune-chemistry experiments and TCGA data [24] to validate the findings. (2) Secondly, in order to study the effect of key gene on the expression of which proteins, we employed the CRISPR [7–9] and RPPA [10–15] biotechnologies to knock out the explored key gene and obtain the quantitative gene/protein data. (3) Thirdly, we used the aforementioned quantitative gene/protein data to develop a precise multi-scale mathematical model to find key proteins, which can be used to investigate the origin of the GBM from the genetic, protein and tissue level starting from explored key gene as well as use the related experiments to evaluate our findings.

In summary, this study has developed an efficient survival analysis algorithm to identify GBM related *Adipocyte enhancer-binding protein 1* (*AEBP1*) gene, and then used the related quantitative gene/protein data to explore the key proteins and molecular mechanism for GBM. Results show significant correlation between *AEBP1* upregulation and increased *EGFR* expression in primary glioma. A glioma cell line LN229 was used to identify major protein players and molecular pathways through RPPA analysis after *AEBP1* overexpression and AEBP1 knockdown. We reveal that *AEBP1* exerts its tumor-promoting effects by mainly activating mTOR pathway in Glioma.

## Methods

**Fig. 1:**
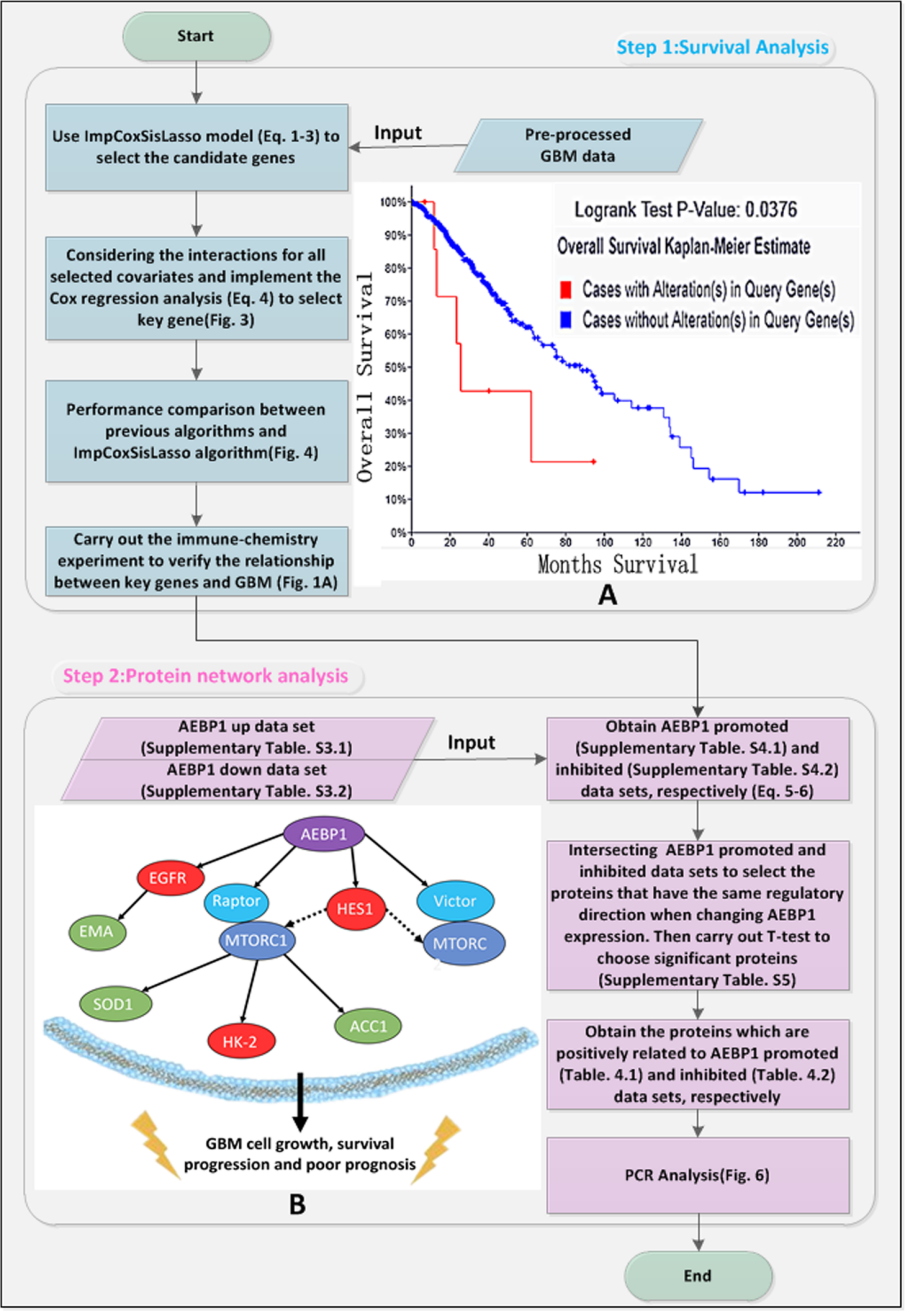
Pipeline of the study. **a** The survival curves for AEBP1 [24]. **b** Schematic diagram illustrating the protein network regulated by AEBP1

The pipeline of the study is illustrated in Fig. 1.

### Survival data

We use a multi-study microarray database of GBM expression profiles (*n* = 227) from the Georgetown Database of Cancer G-DOC [2], based on the Affymetrix U133 plus 2.0 GeneChip microarray platform. The original microarray datasets are normalized and preprocessed by R software package. After preprocessing step, there are 227 samples and 54,675 genes left in the data matrix. Next, the interquartile range (IQR) threshold is employed to screen out the genes with small variance value. After that, there are only 227 samples and 10,992 genes left in the GBM gene expression and survival time data matrix.

### Improved survival analysis algorithm (ImpCoxSisLasso)

**Fig. 2:**
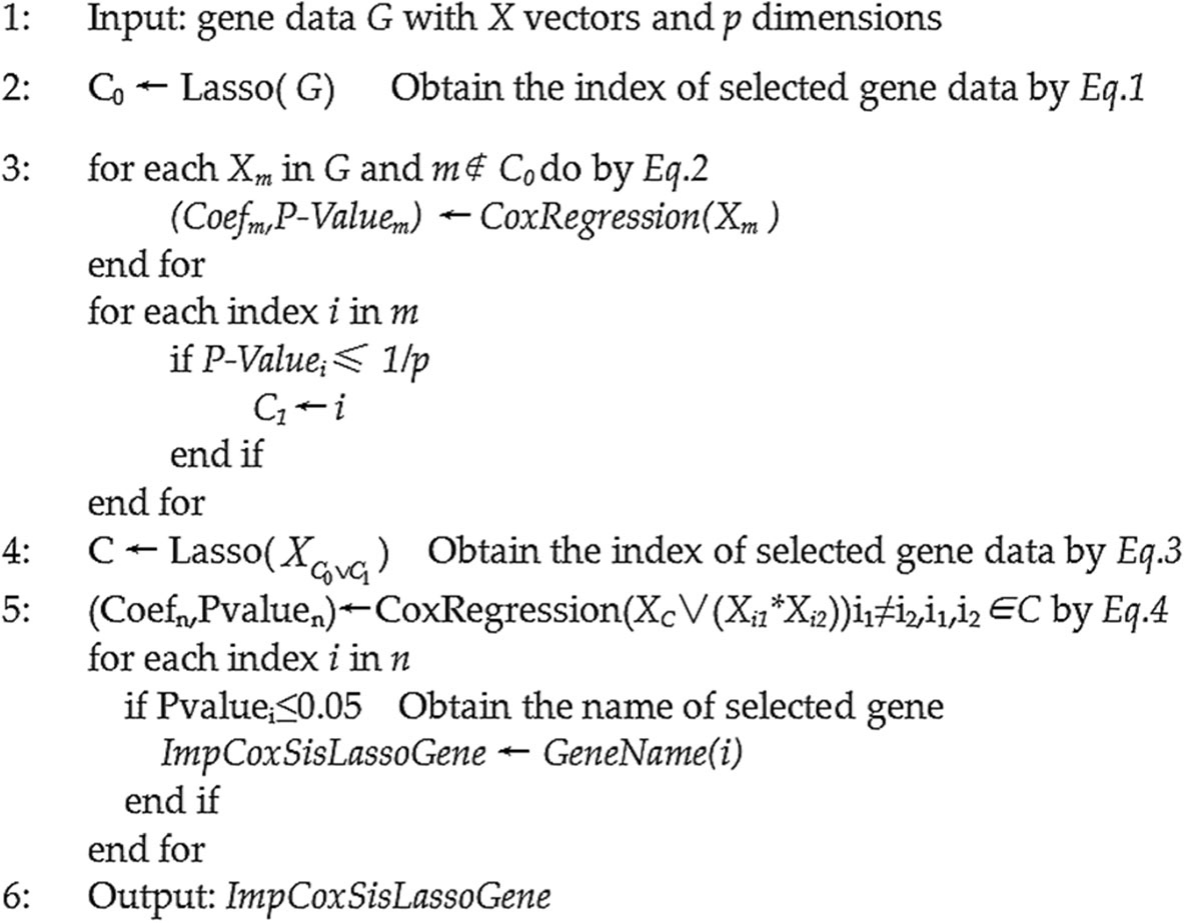
The ImpCoxSisLasso algorithm

We extend our previously well-developed CoxSisLasso strategy [2] by considering the gene interactions to develop ImpCoxSisLasso algorithm(Eq. 1–4).

1 $$ {\hat{\beta}}_{LASSO}=\arg \min \left\{-\sum \limits_{k\in D}\left[{x}_k^T\beta -\log \left({\sum}_{j\in {R}_k}\exp \left({x}_j^T\beta \right)\right)\right]+\lambda \sum \limits_{j=1}^p\left|{\beta}_j\right|\right\} $$

where β is an unknown p-dimensional regression coefficient vector and xi is a vector of potential predictors for the *i*^*th*^ individual. Based on the samples, ${\hat{\beta}}_{LASSO}$ is the LASSO [2] estimator of the unknown parameter coefficients β. D is the set of indices of the events and R_k_ denotes the set of indices of the individuals at risk in time t_k_. k and i represent the index for the set D and set *R*_*k*_, respectively. The tuning parameter λ is used to control the sparsity of the estimator.

2 $$ {\hat{\beta}}_m=\underset{\beta_m}{\arg \max}\left\{\sum \limits_{k\in D}\left[{x}_{k,{C}_0}^T{\beta}_{C_0}+{x}_{k,m}{\beta}_m-\log \left({\sum}_{j\in {R}_k}\exp \left({x}_{j,{C}_0}^T{\beta}_{C_0}+{x}_{j,m}{\beta}_m\right)\right)\right]\right\} $$

where the index of selected covariates for the nonzero components in ${\hat{\beta}}_{LASSO}$ is denoted by C_0_ and each remaining covariate except C_0_ is denoted by x_m_, where m∈{1,2,...,p}. We choose the significant covariates with *P*-Value smaller than a threshold value, 1/p for example.

3 $$ \underset{\beta_{\Theta}}{\min}\left\{-\sum \limits_{k\in D}\left[{x}_{k,\Theta}^T{\beta}_{\Theta}-\log \left({\sum}_{j\in {R}_k}\exp \left({x}_{j,\Theta}^T{\beta}_{\Theta}\right)\right)\right]+\lambda \sum \limits_{j\in {\beta}_{\Theta}}|{\beta}_j|\right\} $$

where Θ is the collection of the augmented selected predictors C_0_∪C_1_ with C_0_ denoting the index of selected covariates with Lasso, and C_1_ denoting the chosen covariates by Eq. 2.

4 $$ \underset{\beta_C,{\beta}_{i_1,{i}_2}}{\max}\sum \limits_{k\in D}\left[\begin{array}{c}{x}_{k,C}^T{\beta}_C+\sum \limits_{i_1\ne {i}_2,{i}_1,{i}_2\in C}{x}_{ki_1}{x}_{ki_2}{\beta}_{i_1,{i}_2}\\ {}-\log \left({\sum}_{j\in {R}_k}\exp \left({x}_{j,C}^T{\beta}_C+\sum \limits_{i_1\ne {i}_2,{i}_1,{i}_2\in C}{x}_{ji_1}{x}_{ji_2}{\beta}_{i_1,{i}_2}\right)\right)\end{array}\right] $$

where C denotes the index of finally selected covariates by Eq. 3. and the product ${x}_{i_1}{x}_{i_2}$ with i_1_ ≠ i_2_, i_1_ and i_2_∈C denote the interactions between the selected genes. Here, i_1_ and i_2_ represent the index for the interactions’ terms. Then, Fig. 2 describes ImpCoxSisLasso algorithm as the following.

### Immune chemistry experiment

Immunofluorescence staining was performed on GBM tissue sections as previously described. The specimens were rapidly isolated and post fixed in 4% PFA for 24 h and then soaked in 40% sucrose for 1 day. GBM tissue sections (10 μm) were obtained using a cryostat (Leica CM3050S-3-1-1, Bannockburn, IL, USA) and permeabilized with 0.3% Triton X-100 in PBS for 30 min. Sections were blocked with 5% donkey serum for 1 h and slices were incubated with a solution of 0.6 mg/ml diaminobenzidine and 0.05% H_2_O_2_ for 2 min. After that, incubation was terminated with three 10-min phosphate-buffered saline washes. Finally, slices were mounted onto gelatin-coated slides and dried overnight before placing a coverslip on them. Representative sections for each GBM tissue were then photographed.

### Cell lines, plasmids

The LN229 cell line was purchased from ATCC [25]. The *pcDNA-AEBP1* Plasmid was provided by Professor Ro from Dalhousie University, Canada [26]. Plasmid for *sgRNA-AEBP1* was constructed by inserting two guide RNA from exon 12 of *AEBP1* into pPB-Cas9-puro at the cloning site. We named the construct with insert as *pPB-Cas9-puro-AEBP1-f1/r4*.

### *AEBP1* overexpression experiment

Cell line LN229 was seeded at 4 × 105 cells/well in a 60 mm plate and incubated in standard culture medium at 370 °C overnight. Culture plates designated for pcDNA vector or pcDNA-AEBP1 transfection were done in triplicates. Transfection was done the next day using Fugene 6 transfection reagent from Roche. We used Reagent to DNA ratio of 3:1 during the transfection. Cells were harvested 48 h after transfection. Growing Cells in culture plates were trypsinized, spun down and washed with PBS for two times. Cell pellet was stored at -80 °C for RPPA analysis. Cells from one plate of vector transfection and another plate of pcDNA-AEBP1 transfection were lysed for Western blotting analysis to confirm the expression of *AEBP1* at the same time of RPPA cell pellet preparation.

### AEBP1 CRISPR-cas9 knockdown experiment

Cell line LN229 was seeded at 4 × 105 cells/well in a 60 mm plate as above. Culture plates designated for vector or sgRNA-AEBP1 transfection were done in triplicates. Transfection was performed the next day after seeding using Fugene 6 transfection reagent from Roche and followed the same procedure as above. 48 h after transfection, puromycin was added to each culture dish at a final concentration of 1.5μg/ml. Transfected LN229 cells survived puromycin selection and grew well. LN229 cell pellet was prepared in the same way as above and stored at -800C for RPPA analysis. RPPA processing of frozen cell pellets: Frozen cell pellets were submitted to the RPPA core facility at M.D. Anderson Cancer Center. At the RPPA core, protein was extracted by applying RPPA lysis buffer to the cell pellets. Protein lysates were serially-diluted in lysis buffer and printed on nitrocellulose-coated RPPA slides. Slides were incubated with around 300 validated primary antibodies followed by binding with corresponding Biotinylated secondary antibodies and Avidin-Biotinylated Peroxidase (Vectastain Elite ABC kit, Vector Lab). Signals were detected by DAB colorimetric reaction. Signals on the slides were scanned and quantified as per protocol at the core facility.

### Polymerase chain reaction (PCR)

Total RNA was extracted from cultured cells using Trizol reagent (Invitrogen, Camarillo, CA, USA). Isolated RNA was reverse-transcribed into cDNA using a cDNA synthesis kit (Vazyme, Jiangsu, China), in accordance with the manufacturer’s protocols. qPCR was performed using synthetic primers and SYBR Green (Thermo, Rockford, IL, USA) with an IQ5 Detection System. After incubating at 50 °C for 2 min and 95 °C for 10 min, the samples were subjected to 40 cycles of 95 °C for 15 s and 60 °C for 1 min. The sequences of the primers specific for target genes are listed in Table 1.

**Table 1:** The sequences of the primers specific for target genes

Target gene	The sequences of the primers specific
HES1-F	5’GGCGGCTAAGGTGTTTGGAGG3’
HES1-R	5’GGGCCGCTGGAAGGTGACAC3’
HK2-F	5’AGGGGACTTTGATATCGACATTG3’
HK2-R	5’GCCCCCCACTCCATATTGATAC3’

## Results

### Key genes selection

**Fig. 3:**
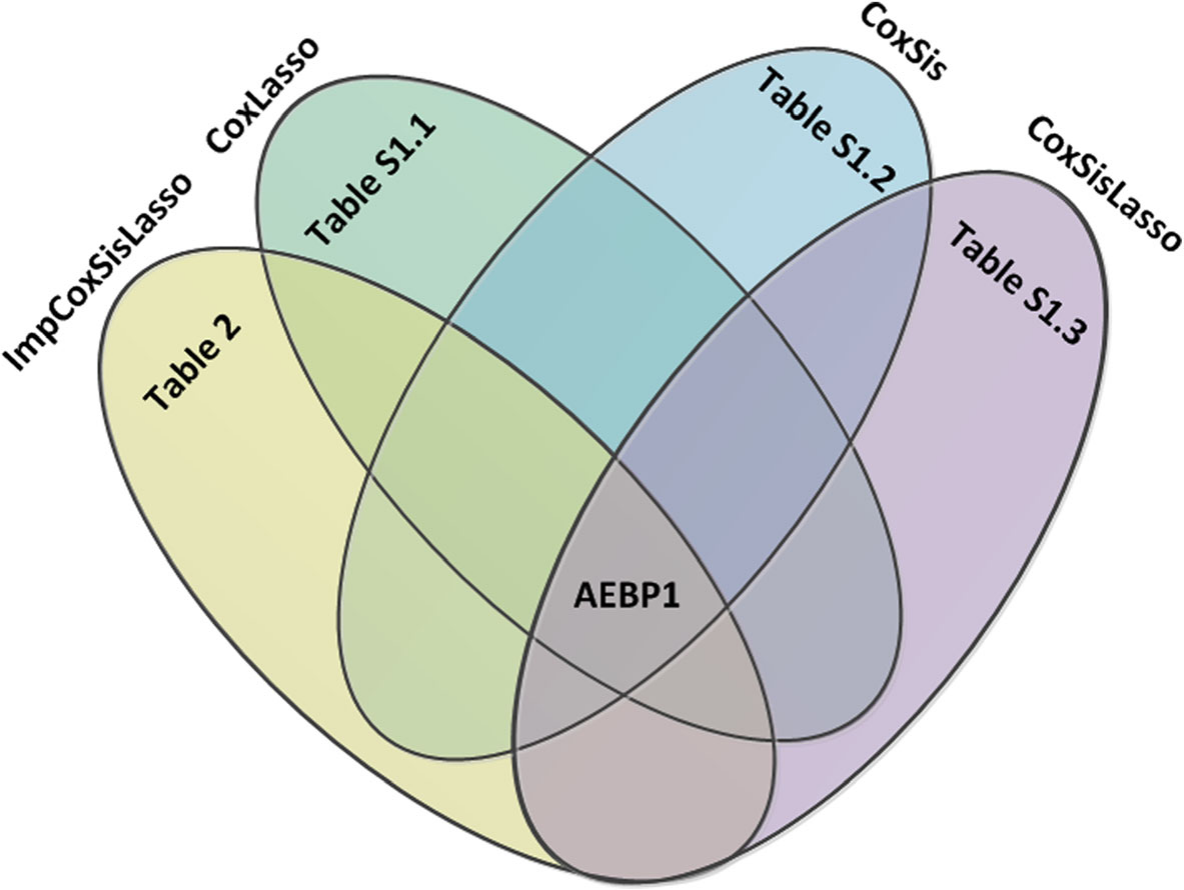
Venn plot for the key gene

By integrating the genes selected by ImpCosSisLasso and previous studies [2], Fig. 3 demonstrates that AEBP1 is the only mutual explored gene for ImpCoxSisLasso, Cox Lasso, Coxsis and CoxSisLasso, which implies that AEBP1 is very potential for the survival time of GBM. The key genes selected by ImpCoxSisLasso are listed in Table 2 and Supplementary Tables S1.1, 1.2, 1.3. The genes selected by previous algorithms [2] are shown in Supplementary Table S1.4.

**Table 2:** Selected genes by ImpCoxSisLasso strategy

Key gene	
AEBP1, CDCA7L, SNTB1, TELO2, SLC35D1, FOXG1, ARIH2_INTS1, ZNF786_AEBP1, ZNF786_INTS1, ZNF786_SGCD, ZNF786_EIF3A, ZNF786_CDCA7L, ZNF786_TELO2, AEBP1_IL17RC, INTS1_SGCD, INTS1_IL17RC, INTS1_SLC35D1, GDNF_IL17RC, SGCD_TELO2, IL17RC_TELO2, CBLN1_TELO2, SLC35D1_TELO2	

### Performance comparison for different survival analysis algorithms

**Fig. 4:**
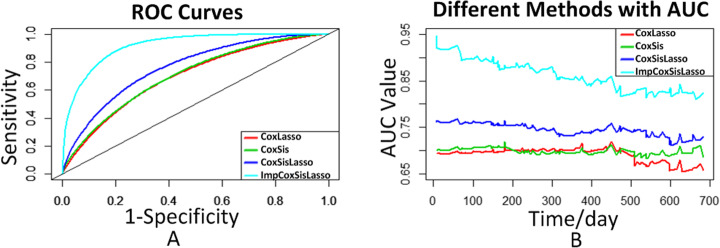
Algorithm performance comparison (**a**) ROC and (**b**) AUC

Here, we use Receiver operating Characteristic (ROC) and Area Under Curve (AUC) [21] curves to compare the performance of the aforementioned algorithms [2]. Both ROC (Fig. 4a) and AUC (Fig. 4b) curves show that ImpCoxSisLasso outperforms the CoxLasso, CoxSis and CoxSisLasso algorithms [2].

### Immune-chemistry experiment

**Fig. 5:**
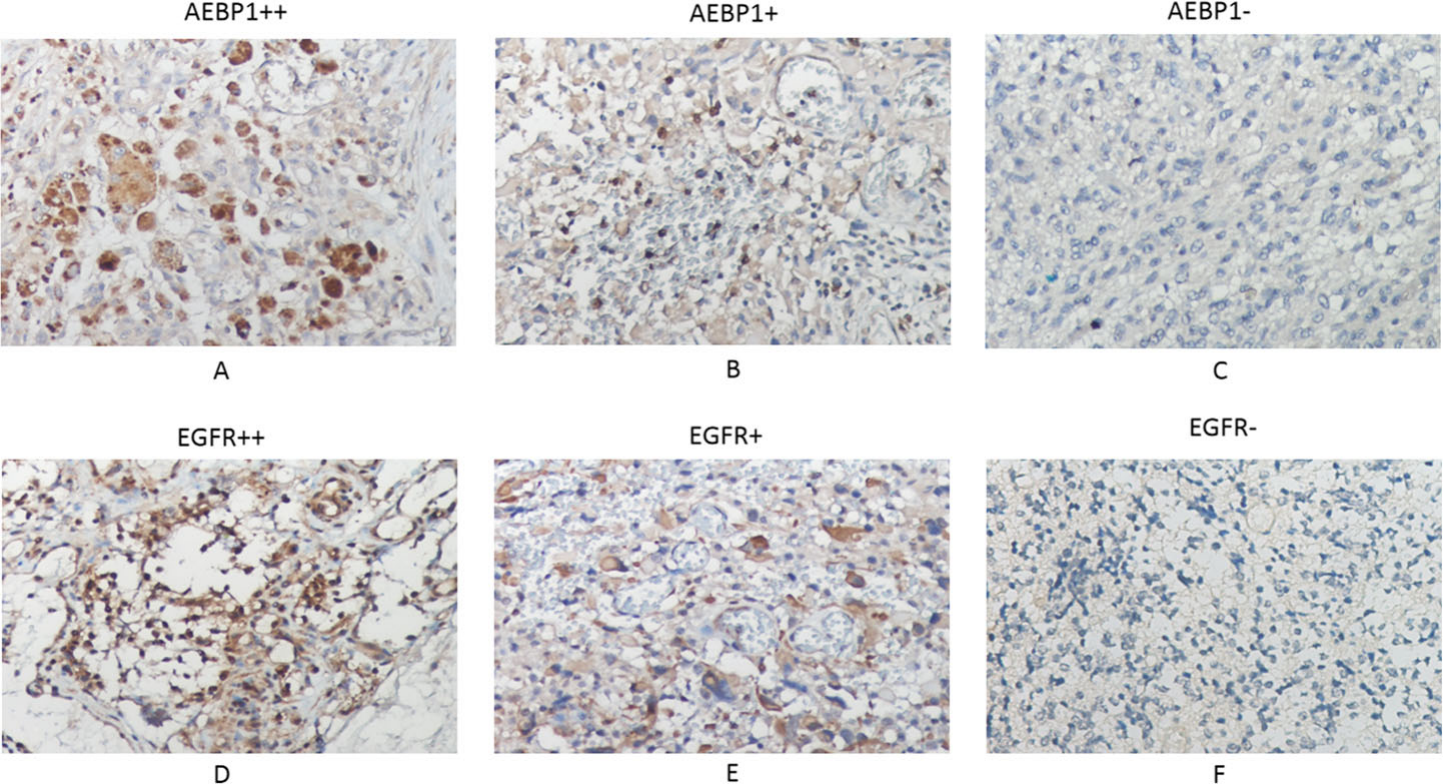
Immunohistochemistry and survival analysis results. A, B and C show high (++), low (+) and negative (−) AEBP1 expression in GBM, respectively. And D, E and F show high (++), low (+) and negative (−) EGFR expression, respectively

Figure 5a, b and c demonstrate that *AEBP1* is positively detectable in GBM. Figure 5d, e and f also show that *EGFR* is positively expressed in GBM. Next, Table 3 shows that there is a strong correlation between *AEBP1* and *EGFR*, since the *p* value of Chi square test [16] is less than 0.05. Lastly, Supplementary Table S2 lists the protein expressions for *AEBP1* and *EGFR* in immune-chemistry experiment, as well as we use Fig. 1a to demonstrate the impact of *AEBP1* on the survival time for GBM patients.

**Table 3:** Statistical analysis of IHC data for AEBP1 and EGFR protein in GBM

	AEBP1 positive (++&+)	AEBP1 negative (−)	Row Total
EGFR+	18	11	29
EGFR-	2	7	9
Column total	20	18	38
*P*-Value	0.036485093		

### Protein network analysis

Firstly, we increase and keep the *AEBP1* expression for LN229 cell lines in the experimental group and control group, respectively. Then, we carry out RPPA experiment twice for these two groups. Finally, the *AEBP1* Up data set (Supplementary Table S3.1) shows 287 related proteins’ expression.

Secondly, we knock out the *AEBP1* by CRISPR-cas9 and keep the *AEBP1* expression for LN229 cell lines in the experimental group and control group, respectively. Then, we carry out RPPA experiment twice for these two groups. Finally, the *AEBP1* Down data set (Supplementary Table S3.2) shows 302 related proteins’ expression.

Thirdly, we use Eq. 5. to compute the fold change between experimental and control group for both AEBP1 Up and Down data set, respectively.

5 $$ {FC}_i=\frac{\sum \limits_{j=1}^m{ExP}_{\mathrm{ij}}}{\sum \limits_{j=1}^m{CoP}_{\mathrm{ij}}} $$

Here, FC_i_ is the fold change between experimental (ExP_ij_) and control group (CoP_ij_). i represents the index of the proteins. j represents the index for the experiment, and m represents the replicates number. ExP_ij_ and CoP_ij_ are the proteins’ expression measured by RPPA experiment.

6 $$ {\mathrm{Judge}}_{\mathrm{i}}\left({\mathrm{FC}}_{\mathrm{i}}\right)=\left\{\begin{array}{c}\mathrm{UP}\kern7.25em {\mathrm{FC}}_{\mathrm{i}}>1\\ {}\mathrm{DOWN}\kern5.25em {\mathrm{FC}}_{\mathrm{i}}\le 1\end{array}\right. $$

Next, we use Eq. 6. to determine which proteins are promoted or inhibited by AEBP1. For *AEBP1* Up data set, the promoted proteins are listed in Supplementary Table S4.1 as AEBP1 promoted data set. For AEBP1 Down data set, the inhibited proteins are listed in Supplementary Table S4.2 as AEBP1 inhibited data set.

Lastly, we compare the experimental group (ExPij) and control group (CoPij) for both AEBP1 Promoted and Inhibited data sets by T test [3]. The null hypothesis is that the average expression level of a protein under experimental condition is equal to the level under control conditions. After we compute the *P*-value for both *AEBP1* promoted data set and *AEBP1* inhibited data set, 7 proteins’ expression are statistically significant in AEBP1 promoted data set (Table 4) and AEBP1 inhibited data set (Table 5), respectively. The detail data is listed in Supplementary Table S5.

**Table 4:** AEBP1 promoted dataset

Protein Name	FC_i_	*P*-value
ACC1-R-C	1.06701864	0.01841297
EMA-M-C	1.36047171	0.02989766
HES1-R-V	1.15596	0.00029086

**Table 5:** AEBP1 inhibited dataset

Protein Name	FC_i_	*P*-value
Hexokinase-II-R-V	0.98022947	0.00639911
Raptor-R-V	0.9132965	0.03564656
Rictor_pT1135-R-V	0.95940456	0.02726222
SOD1-M-V	0.97722104	0.03054562

Since manually reviewed experimental evidences already turn out that *ACC1-R-C* (*ACC1*) [27], *EMA-M-C* (*EMA*) [28], Raptor-R-V (Raptor) [29], *Rictor_pT1135-R-V* (*Rictor*) [30] and *SOD1-M-V* (*SOD1*) [31] are related to *AEBP1*, we then carry out PCR experiment for *HES1-R-V* (*HES1*) and *Hexokinase-II-R-V* (*HK2*) in Cell Line LN-18, LN-229, and U251 to confirm the correlation between the expression of these two proteins and *AEBP1*, respectively.

### PCR verification

**Fig. 6:**
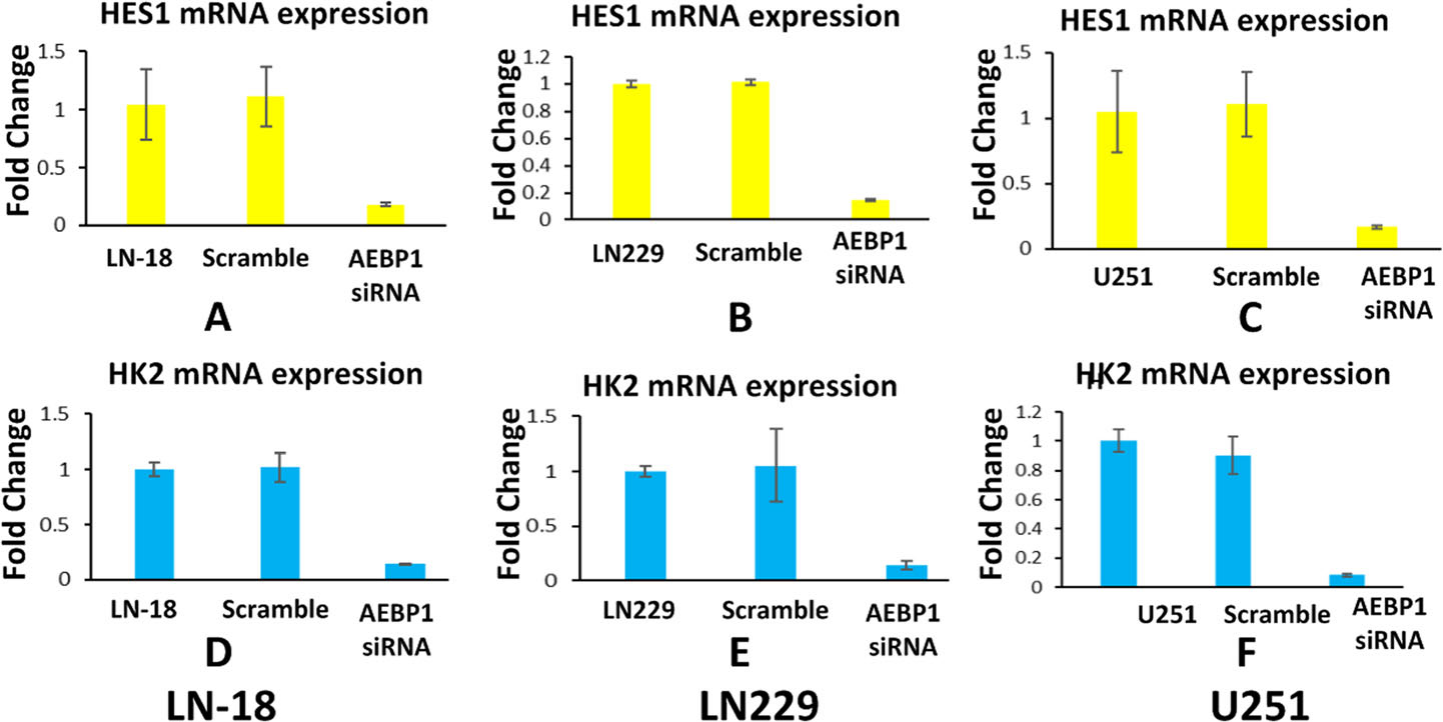
Gene expression of HES1 and HK2 in different cell lines with AEBP1 siRNA knockdown. Here, X-axis represents the cell line, Scramble siRNA and AEBP1 siRNA treatment. Y-axis represents the fold changes of different genes in mRNA levels as a result of AEBP1 knockdown. **a**, **b** and **c** show the HES1 mRNA expression in Cell Line LN-18, LN-229, and U251 respectively. **d**, **e** and **f** show the HK2 mRNA expression in Cell Line LN-18, LN-229, and U251 respectively. Data presented were from qRT-PCR experiment

Figure 6 and Supplementary Table S6 indicate that the expressions of both *HES1* and *HK2* are positively related to the expression of *AEBP1*. Therefore, we conclude that all the proteins listed in Tables 4 and 5 are related to the expression of *AEBP1*.

## Discussion

This study aims to investigate the origin of the GBM from systematic view. Three research questions were proposed and addressed.

Since previous additive survival analysis algorithms (CoxLasso, CoxSis and CoxSisLasso [2]) do not consider the gene’s interaction, they neither agree with the nature of biology [32–35] nor have the high predictive accuracy. Here, after we integrate interaction item into Eq. 4 of ImpCoxSisLasso algorithm, we not only can explore the gene signature of GBM for P> > N type of data (Table 2), but also Fig. 4 turns out that ImpCoxSisLasso algorithm is much better than the previous [2]. Moreover, Fig. 3 shows that both ImpCoxSisLasso algorithm and our previous survival algorithms (CoxLasso, CoxSis and CoxSisLasso [2]) mutually find *AEBP1* as the key gene related to the survival time of GBM patients. Lastly, we use TCGA data [24] (Figs. 1a) and 5 to cross validate the impact of *AEBP1* gene on GBM.

In this report, Table 3 and Fig. 5 demonstrated that there is a significant correlation between the expression of *AEBP1* and *EGFR* in patient samples of GBM. Through overexpression or knockdown of *AEBP1* gene in glioma cell line LN229 and RPPA analysis, we can show that a panel of proteins (Tables 4 and 5) are significantly affected by the manipulation of AEBP1 expression. By a stringent cutoff of *p* < 0.05, *HK2*(Fig. 6), *SOD1* [36], *Raptor* [37], *Rictor* [38] were markedly downregulated when *AEBP1* expression was knocked down. Using the same cutoff standard, *ACC1* [39], *HES1*(Fig. 6) and *EMA* [28] were significantly upregulated when *AEBP1* was overexpressed.

Through extensive literature research [36, 37, 40, 41], we found that affected proteins by either *AEBP1* overexpression or knockdown converge to the regulation of mainly one signaling pathway: the MTOR pathway. MTOR forms two different complexes, MTORC1 and *MTORC2*. *HK2* is a downstream effector of MTORC1 through the transcriptional regulation of transcription factor *HIF1* [41]. It integrates energy metabolism with cancer cell survival. MTORC1 directly regulates *Superoxide dismutase 1* (*SOD1*) through phosphorylation [36]. *SOD1* can in turn act as a nuclear transcription factor to fend off oxidative stress [14] in cancer cells [31]. Raptor is an interacting partner of MTORC1 [40] and phosphorylation of Raptor by MTORC1 is essential for the kinase activity of MTORC1 on its substrates [37]. Dissociation of Raptor from MTORC1 resulted in the inhibition of MTOR signaling activity [29].

On the other hand, *Rictor* is an essential partner of MTORC2 [42, 43]. Blocked association of Rictor from MTORC2 led to the inhibition of MTORC2 activity and cancer cell death in glioma cell line in vitro [30]. In addition, overexpression of Rictor was associated with increased MTORC2 activity and tumor growth in glioma patients [44].

*ACC1* is a *SREBP1* target gene [39]. MTORC1 signaling controls the expression of *SREBP1* and consequently regulates *SREBP1* target gene *ACC1* expression [27]. HES1 (Hairy and Enhancer of Split 1) is a downstream target of Notch signaling pathway [45, 46]. *HES1* antagonizes PTEN-induced inhibition of MTOR signaling activity [47]. *EMA* (Epithelial Membrane Antigen) is a glycosylated protein encoded by *MUC1* gene [28]. Although the cytoplasmic tail of EMA has roles in signal transduction, the relationship between *EMA* and MTOR signaling pathway is not clear at this moment. But *EGFR* signaling activation increased *MUC1* gene expression in some cancer cell lines [48].

Taken together, our results indicate that *AEBP1* regulates the expression of *Raptor* and *Rictor*, key binding partners of MTORC1 and MTORC2. *AEBP1* also has an important role in regulating the expression of *HES1* which promotes MTOR activity by inhibiting PTEN function [47, 49]. Enhanced MTOR signaling by *AEBP1* stimulates downstream expression of MTOR targets *HK2* and *ACC1* or increases phosphorylation of MTOR target *SOD1*. The net effect of *AEBP1* overexpression in glioma is the activation and promotion of MTOR signaling pathway in cancer cells. We illustrate the effects of *AEBP1* with different molecular players on MTOR pathway in a schematic model (Fig. 1b).

*AEBP1* overexpression in glioma is associated with higher tumor grade and worse prognosis [50]. *AEBP1* as therapeutic target for cancer treatment has not drawn much attention. However, clinical trials are under way to test the effectiveness of MTOR inhibitors in the treatment of different types of cancers [51]. Indeed, it has been reported that MTOR inhibitors are effective in a subset of patients with *Rictor* gene amplification in lung cancer [52] and gastric cancer [38]. MTOR inhibitors are actively investigated in preclinical cancer cell line and mouse models of GBM with various success [51, 53]. Experimental results from these preclinical studies place MTOR as the pivotal target in treating GBM [53]. A search of clinical trials in the USA revealed 34 trials using MTOR inhibitors in glioma patients (www.clinicaltrials.gov last accessed on July 20th, 2019). Most of these clinical trials are still under way. Completed trials showed limited successes. In terms of our findings, we may need to take into consideration the expression levels of *AEBP1* in patients’ tumor samples when planning clinical trials of MTOR inhibitors on GBM or evaluating clinical responses of MTOR inhibitors in the treatment of GBM.

## Conclusions

In conclusion, in step one, our improved survival analysis model not only finds that the key gene, but also has better performance and efficiency. In step two, through CRISPR and RPPA techniques, we get reliable protein data, and then search for the seven key proteins by a precise multi-scale mathematical model. And last, we analyze the relationship between AEBP1 and key proteins to identify that AEBP1 exerts its tumor-promoting effects by mainly activating mTOR pathway in Glioma.

Although the results show that we can find the mechanism of AEBP1 in mTOR, we can still improve it. For instance, in protein network, we can consider adding machine learning algorithm to find the key protein. In the future, we can even improve the survival time of patients through the explored pathway mechanism.

## Supplementary information

**Additional file 1.** This file contains all the supplementary tables and figures.

## Data Availability

The datasets supporting the conclusions of this article are included within the article and the additional file.

## Abbreviations

GBM: Glioblastoma multiforme
AEBP1: Adipocyte enhancer-binding protein 1
CRISPR: Clustered regularly interspaced short palindromic repeat sequences
RPPA: Reverse phase protein arrays
IQR: Interquartile range
PCR: Polymerase Chain Reaction
ROC: Receiver operating Characteristic
AUC: Area Under Curve
