# Supplementary material for: Developing the novel bioinformatics algorithms to systematically investigate the connections among survival time, key genes and proteins for Glioblastoma multiforme
Source: BMC Bioinformatics. 2020 Sep 17;21(Suppl 13):383. doi: 10.1186/s12859-020-03674-4 (PMC7646399; doi:10.1186/s12859-020-03674-4)
Supplement: Supplementary file 1 — Additional file 1. This file contains all the supplementary tables and figures. [file 12859_2020_3674_MOESM1_ESM.doc]

**SUPPLEMENTAL INFORMATION**

Supplemental Information includes ten tables.

Supporting Information Legends

# Table:

S1: The selected key genes

S2: Immunohistochemistry experiment results

S3: The related proteins’ expression after we increase or decrease AEBP1 for LN299 cell line

S4: The fold change value of AEBP1 expressed protein.

S5: The P-value of the fold change of protein name by T-test

S6: PCR verification data

**Supplementary tables**

**S1: The selected key genes**

**Table S1.1.** The selected genes for CoxLasso strategy.

| Key gene |
| --- |
| ARIH2, ZNF786, AEBP1, FOXG1, INTS1,GDNF,  CUTC, SGCD, CCM2, IL17RC, EIF3A, CBLN1 |

**Table S1.2.** The selected genes for CoxSis strategy.

| Key gene |
| --- |
| YAP1, TRAF3IP2, AEBP1, GDNF,  EAF2, ST5, IL17RC, EIF3A |

**Table S1.3.** The selected genes for CoxSisLasso strategy.

| Key gene |
| --- |
| ARIH2, ZNF786, AEBP1, FOXG1, INTS1, GDNF,  SGCD, IL17RC, EIF3A, CBLN1, SLC351,  ELOVL2, CDCA7L, SNTB1, TELO2 |

**Table S1.4. The interaction genes of different strategys.**

| Index | Gene Set | Gene |
| --- | --- | --- |
| 1 | ImpCoxSisLasso | ARIH2_INTS1,ZNF786_AEBP1,ZNF786_INTS1,ZNF786_SGCD,ZNF786_EIF3A,ZNF786_CDCA7L,ZNF786_TELO2,AEBP1_IL17RC,INTS1_SGCD,INTS1_IL17RC,INTS1_SLC35D1,GDNF_IL17RC,SGCD_TELO2,IL17RC_TELO2,CBLN1_TELO2,SLC35D1_TELO2 |
| 2 | CoxLasso | CUTC,CCM2 |
| 3 | CoxSis | YAP1,TRAF3IP2,EAF2,ST5 |
| 4 | CoxSisLasso | ELOVL2 |
| 5 | CoxLasso  ImpCoxSisLasso | None |
| 6 | CoxLasso  CoxSis | EIF3A |
| 7 | CoxSis  CoxSisLasso | None |
| 8 | CoxLasso  CoxSisLasso | ARIH2,ZNF786,INTS1,SGCD,CBLN1 |
| 9 | ImpCoxSisLasso  CoxSisLasso | CDCA7L,SNTB1,TELO2,SLC35D1 |
| 10 | ImpCoxSisLasso  CoxSis | None |
| 11 | ImpCoxSisLasso  CoxLasso  CoxSis | None |
| 12 | CoxLasso  CoxSis  CoxSisLasso | GDNF,IL17RC,EIF3A |
| 13 | ImpCoxSisLasso  CoxLasso  CoxSisLasso | FOXG1 |
| 14 | ImpCoxSisLasso  CoxSis  CoxSisLasso | None |
| 15 | ImpCoxSisLasso  CoxLasso  CoxSis  CoxSisLasso | AEBP1 |

**S2: Immunohistochemistry experiment results**

**Table S2.** AEBP1 and EGFR protein expression in GBM by immunohistochemistry (total samples n=38).

| Case ID | AEBP1 | EGFR |
| --- | --- | --- |
| 1420269 | + | + |
| 1421621 | + | +++ |
| 1422237 | - | + |
| 1422453 | + | ++ |
| 1423000 | + | + |
| 1423057 | + | ++ |
| 1423223 | - | - |
| 1425155 | - | ++ |
| 1425254 | + | ++ |
| 1425563 | + | + |
| 1426569 | - | + |
| 1427135 | + | + |
| 1433000 | ++ | ++ |
| 1435026 | ++ | ++ |
| 1436557 | - | + |
| 1502720 | + | - |
| 1502931 | + | ++ |
| 1503194 | - | ++ |
| 1503468 | - | + |
| 1503820 | + | + |
| 12080 | + | ++ |
| 410053 | + | + |
| 410442 | - | - |
| 411921 | - | - |
| 511549 | - | + |
| 607799 | - | + |
| 611724 | - | - |
| 714848 | - | + |
| 812744 | - | - |
| 910530 | - | + |
| 916026 | ++ | ++ |
| 916049 | + | ++ |
| 917181 | - | - |
| 917283 | - | ++ |
| 918283 | + | ++ |
| 918464 | + | - |
| 918925 | ++ | + |
| 1101979 | + | ++ |

Here, ++, +, and - represent high, low and negative gene expression in GBM, respectively.

**S3:** The related proteins’ expression after we increase or decrease AEBP1 for LN299 cell line

**Table S3.1.** The data of proteins expression of AEBP1 high expression in LN229

The data is listed on <https://github.com/347251369/supplementary-of-AEBP1> **Supplementary Table S3.1.xlsx**

**Table S3.2.** The data of proteins expression of AEBP1 negative expression in LN229

The data is listed on <https://github.com/347251369/supplementary-of-AEBP1> **Supplementary Table S3.2.xlsx**

**S4: The fold change value of AEBP1 expressed protein.**

**Table S4.1.** The AEBP1 promoted data set

The data is listed on <https://github.com/347251369/supplementary-of-AEBP1> **Supplementary Table S4.1.xlsx**

**Table S4.2.** The AEBP1 inhibited data set

The data is listed on <https://github.com/347251369/supplementary-of-AEBP1> **Supplementary Table S4.2.xlsx**

**S5: The P-value of the fold change of protein name by T-test**

**Table S5.** The P-value of the fold change of protein name by T-test

|  | AEBP1 promoted data set | | AEBP1 inhibited data set | |
| --- | --- | --- | --- | --- |
| Protein name | fold change | P-value | Fold change | P-value |
| ACC_pS79-R-V | 1.012158456 | 0.896248976 | 0.869396375 | 0.284785483 |
| ACC1-R-C | 1.067018635 | 0.018412974 | 0.936080294 | 0.537003867 |
| ADAR1-M-V | 1.043046642 | 0.73268395 | 0.911251078 | 0.24863322 |
| AMPKa-R-C | 1.010277618 | 0.52935455 | 0.992958401 | 0.851890619 |
| Annexin-I-M-V | 1.009337224 | 0.847398336 | 0.881453308 | 0.547496411 |
| A-Raf-R-V | 1.006918969 | 0.96815373 | 0.974855157 | 0.565137226 |
| AR-R-V | 1.015351757 | 0.432081742 | 0.98736398 | 0.822133144 |
| ATM_pS1981-R-V | 1.008331583 | 0.764210708 | 0.99139629 | 0.815367676 |
| ATM-R-V | 1.032137536 | 0.83301984 | 0.889482718 | 0.220709856 |
| B7-H4-R-C | 1.025900156 | 0.751660377 | 0.994003982 | 0.844006038 |
| Bad_pS112-R-V | 1.023769654 | 0.776802908 | 0.949709572 | 0.10777971 |
| Bak-R-C | 1.077072611 | 0.155068957 | 0.986570568 | 0.713114471 |
| b-Catenin-R-V | 1.01936637 | 0.878427427 | 0.905636086 | 0.39505047 |
| Bid-R-C | 1.005382077 | 0.910164856 | 0.992738683 | 0.865892704 |
| Bim-R-V | 1.026700059 | 0.913949017 | 0.983778937 | 0.840821151 |
| BiP-GRP78-M-C | 1.035866235 | 0.785037697 | 0.932962288 | 0.583942907 |
| B-Raf_pS445-R-V | 1.01320457 | 0.893331022 | 0.949655835 | 0.359273394 |
| CD31-M-V | 1.013766518 | 0.857005567 | 0.985320482 | 0.750387211 |
| Chk1_pS296-R-V | 1.000980089 | 0.995957322 | 0.981255256 | 0.276916167 |
| c-Kit-R-V | 1.017307266 | 0.689006705 | 0.98624931 | 0.490030134 |
| Collagen-VI-R-V | 1.004095757 | 0.958806333 | 0.933418461 | 0.738157563 |
| Cox2-R-C | 1.028878402 | 0.902693411 | 0.967537519 | 0.328012449 |
| C-Raf_pS338-R-V | 1.040777274 | 0.055531653 | 0.990670752 | 0.533776097 |
| Cyclin-B1-R-V | 1.02936668 | 0.449170927 | 0.98071873 | 0.713565782 |
| Cyclin-D1-R-V | 1.047708221 | 0.095629071 | 0.989609232 | 0.868947082 |
| Cyclin-E1-M-V | 1.010203611 | 0.840070847 | 0.918900296 | 0.155516481 |
| Cyclophilin-F-M-V | 1.001134788 | 0.951617489 | 0.929413766 | 0.56236766 |
| DM-K9-Histone-H3-R-C | 1.035175739 | 0.111017828 | 0.981540612 | 0.774792094 |
| E-Cadherin-R-V | 1.081375782 | 0.425962984 | 0.996259177 | 0.938455982 |
| EGFR_pY1173-R-V | 1.018915775 | 0.524943715 | 0.966034426 | 0.434844952 |
| EMA-M-C | 1.36047171 | 0.029897664 | 0.973634214 | 0.795212242 |
| ERCC5-R-C | 1.00948112 | 0.923717219 | 0.968391959 | 0.613986982 |
| FASN-R-V | 1.013853448 | 0.932774358 | 0.934558355 | 0.105182046 |
| FoxM1-R-V | 1.0262126 | 0.502880601 | 0.942635843 | 0.07065846 |
| GSK-3a-b-M-V | 1.035655482 | 0.676785225 | 0.962225529 | 0.187609853 |
| H2AX_pS140-M-C | 1.01081707 | 0.792169455 | 0.958739703 | 0.630339528 |
| HER3_pY1289-R-C | 1.018286753 | 0.528002985 | 0.977419953 | 0.433428761 |
| HES1-R-V | 1.155960005 | 0.000290858 | 0.993088231 | 0.840859083 |
| Hexokinase-II-R-V | 1.088278433 | 0.620002048 | 0.980229468 | 0.006399113 |
| IGFBP2-R-V | 1.033772819 | 0.620486816 | 0.973517229 | 0.543700839 |
| JNK_pT183_Y185-R-V | 1.024640605 | 0.635130149 | 0.938846927 | 0.442408166 |
| LC3A-B-R-C | 1.016162626 | 0.88292685 | 0.983238938 | 0.853487383 |
| LDHA-R-C | 1.009708986 | 0.76772627 | 0.922932738 | 0.411284358 |
| MIG6-M-V | 1.014286708 | 0.87898108 | 0.957931527 | 0.067862044 |
| mTOR-R-V | 1.01465779 | 0.773509649 | 0.934310999 | 0.258298285 |
| Myosin-11-R-V | 1.031934869 | 0.765258133 | 0.989442436 | 0.653324687 |
| NAPSIN-A-R-C | 1.006315681 | 0.634005097 | 0.998786319 | 0.964941727 |
| Notch3-R-C | 1.020963102 | 0.94373949 | 0.954296941 | 0.558257109 |
| Oct-4-R-C | 1.004894708 | 0.929117946 | 0.942078287 | 0.308738498 |
| p16INK4a-R-V | 1.078275567 | 0.238567708 | 0.971408548 | 0.372260529 |
| p21-R-V | 1.006144298 | 0.897245439 | 0.976149253 | 0.730526017 |
| p38_pT180_Y182-R-V | 1.021616859 | 0.920165623 | 0.774699483 | 0.111799442 |
| p38-MAPK-R-V | 1.000235178 | 0.994718322 | 0.991141501 | 0.891198392 |
| p70-S6K1-R-V | 1.005817267 | 0.934005946 | 0.987794389 | 0.782730549 |
| PAICS-R-C | 1.035775206 | 0.383463592 | 0.985993111 | 0.759500762 |
| PDHK1-R-C | 1.017555709 | 0.73076088 | 0.972518534 | 0.491227178 |
| PI3K-p110-a-R-C | 1.031034902 | 0.756828226 | 0.99293018 | 0.057030029 |
| PI3K-p110-b-M-C | 1.004917543 | 0.980913617 | 0.962275673 | 0.386851699 |
| PKA-a-R-V | 1.022163773 | 0.798367889 | 0.858626264 | 0.285991561 |
| PKCa-R-V | 1.037300416 | 0.420852947 | 0.923333885 | 0.25257081 |
| PKC-b-II_pS660-R-V | 1.018898622 | 0.76277304 | 0.975446484 | 0.516366202 |
| PKM2-R-C | 1.009897903 | 0.651877413 | 0.901987357 | 0.099371624 |
| PLC-gamma2_pY759-R-C | 1.01295396 | 0.594576301 | 0.994555935 | 0.892474276 |
| PREX1-R-V | 1.010525862 | 0.927005193 | 0.911220839 | 0.384420736 |
| Rad50-M-V | 1.040105799 | 0.558177006 | 0.897267211 | 0.457392417 |
| Raptor-R-V | 1.00800655 | 0.86605101 | 0.913296503 | 0.03564656 |
| Rheb-M-C | 1.042335103 | 0.675076044 | 0.949112116 | 0.416040809 |
| Rictor_pT1135-R-V | 1.078021616 | 0.395756001 | 0.959404564 | 0.027262225 |
| RIP-R-C | 1.00001408 | 0.999847481 | 0.989793319 | 0.811531333 |
| SCD-M-V | 1.000069701 | 0.993922722 | 0.986380452 | 0.448397072 |
| Smac-M-Q | 1.075142641 | 0.390918523 | 0.990176442 | 0.810328891 |
| Smad1-R-V | 1.008147968 | 0.871341035 | 0.97250479 | 0.313168815 |
| Snail-M-Q | 1.071827822 | 0.535259272 | 0.888031401 | 0.15060832 |
| SOD1-M-V | 1.018371818 | 0.667781883 | 0.977221036 | 0.030545618 |
| SOD2-R-V | 1.123384277 | 0.713113444 | 0.898698275 | 0.257078819 |
| Src_pY527-R-V | 1.000481521 | 0.997660462 | 0.999300424 | 0.987799989 |
| Src-M-V | 1.018127183 | 0.709927042 | 0.981750566 | 0.762528828 |
| Stat5a-R-V | 1.028012998 | 0.713105007 | 0.931617008 | 0.440071288 |
| TAZ-R-V | 1.008386974 | 0.955124659 | 0.903190557 | 0.428774823 |
| TSC1-R-C | 1.029028821 | 0.321300047 | 0.983723391 | 0.650338112 |
| Tuberin-R-V | 1.031585576 | 0.556937236 | 0.973328279 | 0.379877049 |
| Tyro3-R-V | 1.037261832 | 0.259430936 | 0.962519796 | 0.149026934 |
| UBAC1-R-V | 1.001019493 | 0.978479023 | 0.987768836 | 0.820788766 |
| ULK1_pS757-R-C | 1.036783556 | 0.672686857 | 0.886499367 | 0.115839456 |
| WIPI1-R-C | 1.01067311 | 0.93355524 | 0.933129837 | 0.116633141 |
| XPA-M-V | 1.029428128 | 0.773546312 | 0.978336136 | 0.154526419 |

**S6: PCR verification data**

**Table S6.** The Hes1 mRNA expression data and HK2 mRNA expression data

|  |  | Cell Line | | | | | | | | |
| --- | --- | --- | --- | --- | --- | --- | --- | --- | --- | --- |
|  |  | LN-18 | | | LN229 | | | U251 | | |
| mRNA |  | LN-18 | Scramble | AEBP1 siRNA | LN229 | Scramble | AEBP1 siRNA | U251 | Scramble | AEBP1 siRNA |
| Hes1 | Mean | 1.048 | 1.116 | 0.179 | 1.000 | 1.013 | 0.143 | 1.048 | 1.107 | 0.169 |
|  | STDEV | 0.304 | 0.256 | 0.144 | 0.027 | 0.021 | 0.008 | 0.314 | 0.248 | 0.013 |
| HK2 | Mean | 1.002 | 1.019 | 0.139 | 1.000 | 1.052 | 0.138 | 1.003 | 0.904 | 0.083 |
|  | STDEV | 0.065 | 0.130 | 0.014 | 0.046 | 0.330 | 0.039 | 0.080 | 0.129 | 0.012 |
